# Comparative transcriptome analyses of genes involved in sulforaphane metabolism at different treatment in Chinese kale using full-length transcriptome sequencing

**DOI:** 10.1186/s12864-019-5758-2

**Published:** 2019-05-14

**Authors:** Qiuyun Wu, Junwei Wang, Shuxiang Mao, Haoran Xu, Qi Wu, Mantian Liang, Yiming Yuan, Mingyue Liu, Ke Huang

**Affiliations:** College of Horticulture and Landscape, Hunan Agricultural University, No.1 Nongda Road, Furong District, Changsha, 410128 Hunan Province China

**Keywords:** Single-molecule real-time sequencing, Next-generation sequencing, Chinese kale, Sulforaphane, De novo assembly

## Abstract

**Background:**

Sulforaphane is a natural isothiocyanate available from cruciferous vegetables with multiple characteristics including antioxidant, antitumor and anti-inflammatory effect. Single-molecule real-time (SMRT) sequencing has been used for long-read de novo assembly of plant genome. Here, we investigated the molecular mechanism related to glucosinolates biosynthesis in Chinese kale using combined NGS and SMRT sequencing.

**Results:**

SMRT sequencing produced 185,134 unigenes, higher than 129,325 in next-generation sequencing (NGS). NaCl (75 mM), methyl jasmonate (MeJA, 40 μM), selenate (Se, sodium selenite 100 μM), and brassinolide (BR, 1.5 μM) treatment induced 6893, 13,287, 13,659 and 11,041 differentially expressed genes (DEGs) in Chinese kale seedlings comparing with control. These genes were associated with pathways of glucosinolates biosynthesis, including phenylalanine, tyrosine and tryptophan biosynthesis, cysteine and methionine metabolism, and glucosinolate biosynthesis. We found NaCl decreased sulforaphane and glucosinolates (indolic and aliphatic) contents and downregulated expression of cytochrome P45083b1 (CYP83b1), S-alkyl-thiohydroximatelyase or carbon–sulfur lyase (SUR1) and UDP-glycosyltransferase 74B1 (UGT74b1). MeJA increased sulforaphane and glucosinolates contents and upregulated the expression of CYP83b1, SUR1 and UGT74b1; Se increased sulforaphane; BR increased expression of CYP83b1, SUR1 and UGT74b1, and increased glucosinolates contents. The desulfoglucosinolate sulfotransferases ST5a_b_c were decreased by all treatments.

**Conclusions:**

We confirmed that NaCl inhibited the biosynthesis of both indolic and aliphatic glucosinolates, while MeJA and BR increased them. MeJA and BR treatments, conferred the biosynthesis of glucosinolates, and Se and MeJA contributed to sulforaphane in Chinese kale via regulating the expression of CYP83b1, SUR1 and UGT74b1.

**Electronic supplementary material:**

The online version of this article (10.1186/s12864-019-5758-2) contains supplementary material, which is available to authorized users.

## Highlights

1. NaCl reduced sulforaphane and glucosinolate contents in Chinese kale.
2. MeJA, BR, and Se increased sulforaphane and/or glucosinolate contents.
3. SMRT sequencing generated 185,134 unigenes, higher than 129,325 in NGS.
4. Combined NGS/SMRT sequencing identified DEGs, CYP83b1, SUR1, and UGT74b1.

## Background

Sulforaphane is a natural isothiocyanate available from widely consumed cruciferous vegetables, including broccoli, radish, and kale. Multiple characteristics of sulforaphane have been widely reported during the past two decades. Sulforaphane has various effects including antioxidant, antitumor formation and anti-inflammatory effect [1–5]. This compound has been shown to suppress or prevent tumor formation and development [2, 5].

One reason of for the great interest on sulforaphane is that it is an inducer of phase 2 enzymes, including phase 2 detoxification enzymes glucuronyltransferase (GT) and NAD(P)H:quinone reductase 1 (NQO1) [2, 6, 7]. Moreover, sulforaphane could stimulate the accumulation of glutathione, which plays important roles in cells including antioxidation, immune response, detoxification, and cancer pathogenesis [8]. For instance, sulforaphane has been reported to induce apoptosis and cell cycle arrest of human colon cancer cells by increasing cyclins A and B1 expression [2]; the administration of sulforaphane shows antitumor formation [9]; sulforaphane inhibits the Aβ1–42-induced excessive secretion of interleukin-1β (IL-1β) in human microglia-like cells via activation of nuclear factor erythroid 2-related factor 2 (Nrf2), a bZIP transcription factor and an inducer of phase 2 genes [10].

Sulforaphane, 1-isothiocyanato-4-(methyl-sulfinyl) butane, is a hydrolysis product of glucosinolate, 4-(methylsulfinyl)butyl glucosinolate, to be exact [11, 12]. Glucosinolate and its hydrolysis products, including isothiocyanates, are the secondary metabolites in plants, especially in various kinds of edible cruciferous vegetables [11]. The formation of glucosinolate is an enzymatic reaction, including cytochrome P450 monooxygenases, methylthioalkylmalate synthases (MAMs),S-alkyl-thiohydroximatelyase or carbon–sulfur lyase (SUR1) [13, 14]. The biosynthesis and accumulation of glucosinolates as well as sulforaphane is influenced by temperature, processing methods and abiotic stresses [11, 15–18]. Esfandiari et al. showed that NaCl treatment (80 and 160 mM) decreased the contents of glucosinolates (including gluconapin, 4-methoxyglucobrassicin and neoglucobrassicin) and increased sulforaphane, and the administration of salicylic acid (SA, 100 μM) lowered 80 mM NaCl-reduced gluconapin in *Brassica oleracea* [17]. They confirmed that NaCl and SA co-treatment induced sulforaphane and isothiocyanates accumulation was independent of the biosynthetic genes of cytochrome P45083b1 (CYP83b1), CYP83a1 and CYP79b2, but associated with the increased hydrolytic enzymes of myrosinase (MYO) and epithiospecifier moifier 1 (ESM1) [17]. Other researches showed the indole-3-acetic acid, methyl jasmonate (MeJA) and brassinosteroids treatments upregulated the production of glucosinolates [19, 20],whereas the contents of glucosinolates could be decreased under selenium (Se) stress [21, 22]. Tian et al. showed the expression of CYP79b2, CYP83b1, and CYP83a1 were significantly downregulated in *B. oleracea* following Se treatment [22]. Ku et al. performed a comparative transcriptome analysis in two broccoli cultivars under MeJA treatment and identified that the glucosinolates’ core structure biosynthesis genes (including CYP79b2, UDP-glycosyltransferase 74B1, UGT74b1, SUR1) were upregulated by MeJA [23]. Several comparative transcriptome analyses in Chinese kale (*B. oleracea* var. *alboglabra* Bailey) [24, 25] and watercress [26] showed that the expression patterns of aforementioned genes related to glucosinolates biosynthesis were changed in different tissues. These reports demonstrate the complex and diversity glucosinolates biosynthesis and metabolism by different stresses and in different plants. It has been reported that the influence of stress-induced biosynthesis of sulforaphane is dependent on plant variety and growth conditions [17, 27]. However, the causality of abiotic stresses-induced dysregulation of sulforaphane and glucosinolates biosynthesis has yet to be clearly explored, and the underlying mechanisms should be dissected.

In comparison with the next (second)-generation sequencing (NGS), the third-generation transcriptome (single-molecule real-time, SMRT) sequencing produced more complete transcriptome data with longer reads. SMRT sequencing has been widely used for plant genome assembly [28]. The problem of higher error rate in SMRT sequencing data could be resulted by correcting with NGS-generated short reads. Here we investigated the molecular mechanisms related to glucosinolates biosynthesis under different abiotic stresses in Chinese kale using combined NGS and SMRT sequencing data. Chine kale is a native Chinese brassica vegetable with good flavor and high content of vitamin C, total phenolics, and glucosinolates. The market demand of Chinese kale sprouts is increasing due to the rich in glucosinolates. Transcriptome with complete and full-length reads would be to dissect molecular mechanisms of glucosinolates biosynthesis in Chinese kale.

## Results

### Sulforaphane and glucosinolates contents were changed by different treatments

**Fig. 1:**
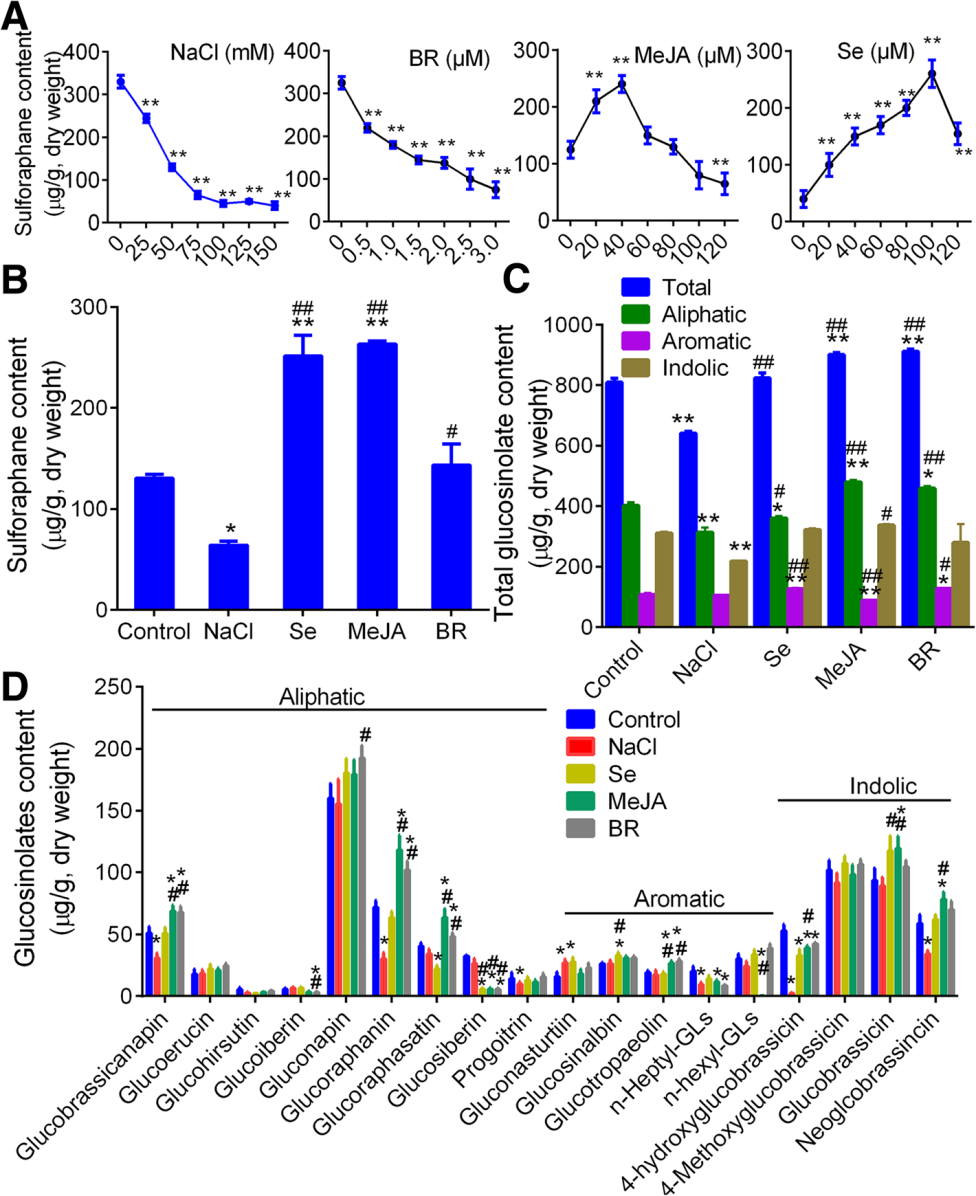
The content of sulforaphane and glucosinolates in Chinese kale seedlings in response to different treatments. **a** the content of sulforaphane in Chinese kale seedlings under different treatments with series concentrations. **b** the content of sulforaphane under different treatments with optimal contents. **c** the total content of 18, aliphatic, aromatic and indolic glucosinolates in Chinese kale seedlings (dry weight, g). **d** the content of 18 glucosinolates by ULPC-MS methods. MeJA, methyl jasmonate. Se, sodium selenite. BR, brassinolide. GLs, glucosinolates. In Fig. 3a and b, * and ** notes *p* < 0.05 and 0.01 vs. control, respectively, while # and ## notes *p* < 0.05 and *p* < 0.01 vs. NaCl, respectively. In Fig. C, * notes *p* < 0.05 vs. control, and # notes *p* < 0.05 vs. NaCl, respectively

We firstly treated with Chinese kale seedlings with series of NaCl, Se, MeJA and BR to select the optimal concentrations. We confirmed all treatments induced dose-dependent increment or reduction in sulforaphane contents (Fig. 1a). Treatment with NaCl (0–150 mM) and BR (0–3.0 μM) significantly reduced sulforaphane. Lower concentrations of MeJA (0–40 μM) and Se (0–100 μM) increased sulforaphane, while overdose MeJA (> 40 μM) and Se (> 100 μM) decreased sulforaphane. We chose 75 mM NaCl, 1.5 μM BR, 40 μM MeJA and 100 μM Se as optimal concentrations for the further treatments.

We confirmed that the contents of sulforaphane (Fig. 1b) and glucosinolates (Fig. 1c, total, aliphatic and indolic) in Chinese kale seedlings were significantly reduced by NaCl treatment. Se and MeJA treatments obviously increased the contents of sulforaphane in kale seedlings in comparison with control (*p* < 0.01; Fig. 3a), and MeJA and BR treatments significantly increased the contents of total and aliphatic glucosinolates (*p* < 0.01, Fig. 1c). The content of aromatic glucosinolates was only increased by Se treatment (*p* < 0.01) compared with control, while MeJA increased both aliphatic and indolic glucosinolates, but decreased aromatic glucosinolates (*p* < 0.01, Fig. 1c). Figure 1d shows the influence of four treatments on the accumulation of 18 glucosinolates. We observed that BR showed slight influence on indolic glucosinolates, followed by aromatic glucosinolates and significantly obvious influence on aliphatic glucosinolates, including gluconapin, glucobrassicanapin, and glucoraphasatin (Fig. 1d); and MeJA showed obvious influence on most glucosinolates, including glucobrassicanapin, glucoraphanin, glucoraphasatin, n-hexyl- glucosinolates and glucobrassicin (*p* < 0.05).

### Summary of Illumina Hiseq and PacBio RSII transcriptome sequencing data of Chinese kale

Illumina Hiseq 2500 platform generated 816,939,038 raw reads and 799,683,878 clean reads (97.89%, 119.97 G) with an average Q_30_value of 93.46% and an average GC content of 47.05% (Table 1). A total of 130,553 unigenes with a 99.05% annotation rate in at least one database [NR (NCBI non-redundant protein sequences), NT (NCBI non-redundant nucleotide sequences), Swissprot, Protein family (Pfam), Gene Ontology (GO), KOG (euKaryotic Orthologous Groups), KEGG (Kyoto Encyclopedia of Genes and Genomes) and KO (KEGG Ortholog database)], including 8006 transcription factors (TFs) were identified from Illumina transcriptome (Table 2). PacBio RSII platform generated a total of 617,063 circular consensus sequences (CCSs) with a full length of 467,672 bp. The full-length non chimera (FLNC) reads number was 434,967, with an average length of 1720 bp (Table 2). PacBio RSII platform produced a total of 199,106 consensus reads and 14,267,901 subreads (13.46 G bases, with an average length of 944 bp, and an N50 of 1572 bp), which were then corrected using the Illumina reads (Additional file 1: Figure S1). The length distributions of gene (Additional file 2: Figure S2A and B), corrected subreads and consensus reads (Additional file 2: Figure S2C), lncRNA number (Additional file 2: Figure S2 S2D) and simple sequence repeat (SSR) motifs (Additional file 2: Figure S2E) are shown in Additional file 2: Figure S2. There was similar distribution of CDS of these unigenes produced by PacBio RSII platform and Illumina Hiseq 2500 platform, and most CDSs were less than 1800 bp (Additional file 2: Figure S2A and B). A total of 6369 long non-coding RNAs (lncRNAs) were identified from PacBio transcriptome (Additional file 2: Figure S2D).

**Table 1:** Summary of the transcriptome data from PacBio RSII platform and Illumina Hiseq platform

Platform	
Illumina Hiseq (*n* = 15)	
Raw reads (average)	5,562,602.5
Clean reads (average)	53,312,258.5
Clean bases (G, total)	119.97
Base error rate (average)	0.015%
Q30 (average)	93.46%
GC content (average)	47.05%
Unigenes annotated in at least one database (NR, NT, KO, Swissprot, Pfam, GO, KOG)	129,325
Unigenes annotated in all databeses	29,191
Total unigenes	130,553
TF number	8006 (6.19%)
PacBio RSII (*n* = 15)	
Total CCS	617,063
Full length reads	467,672 (75.9%)
FLNC reads	434,967 (70.5%)
Average FLNC read length	1720 bp
Consensus reads	199,106
Subreads base (G)	13.46
Subreads number	14,267,901
Average subreads length	944 bp
N50	1572
Total unigenes annotated in at least one database (NR, NT, KOG, Swissprot, Pfam, GO, KEGG)	140,196
Total unigenes	44,938
TF number	9407 (5.08%)

*CCS* circular consensus sequences, *FLNC* full-length non chimera, *GO* Gene Ontology, *KEGG* Kyoto Encyclopedia of Genes and Genomes, *KO* KEGG Ortholog database, *KOG* euKaryotic Orthologous Groups, *Nr* NCBI non-redundant protein sequences, *Nt* NCBI non-redundant nucleotide sequences, *Pfam* Protein family, *TF* transcription factors

**Table 2:** The primers’ sequences of 22 genes for PCR analysis in this study

Genes ID	Gene and symbol	Primers sequences (5′-3′)	
		Forward	Reverse
CDX99400.1	(cytosine-5)-methyltransferase CMT2 (DNMT2)	GAACGACGATGCTGAAGGTAG	CTGCTGCTGAGACTCTGACTA
CDY40532.1	S-adenosylmethionine synthase (SAMS)	AAGCCGCCACATAAGAGACTA	ACAGGACAAGAACAGACAGACT
XP_009111749.1	Uncharacterized	CCTGCCGTGTTGTCTTCC	GCTCTGTGGTTGTTCATCATCT
CDY19364.1	(cytosine-5)-methyltransferase CMT1 (DNMT1)	TGGATGGTGGAGATGCTTCA	CGTTCAAGGTCGTCGTCATT
CDX85436.1	5-methyltetrahydropteroyltriglutamate--homocysteine methyltransferase 1 (metE)	ATGCTCGGTGCTGTTCCA	GCCTTGTGCGATGCGTAA
CDX80735.1	cytochrome P450 83B1 (CYP83b1)	TTCACCGCTCGTCCTCTC	ACCATACACATCTTCCTCATCTCA
CDX69373.1	cytochrome P450 79B1 (CYP79b1)	TTCGGATCTCACTTCCACTACA	CATCACCAAGGTTATAGCCACAA
CDY01177.1	adenosylhomocysteinase 1 (SAHH)	CCGTCACCAAGAGCAAGTTC	CATCACCATATCCGCAGACAAC
XM_009134853.1	S-adenosylmethionine synthase 3-like (SAMS3L)	GCACGCCGCTGTATAGTC	ACACGAGTATATCCTTGTCTGGTA
XP_009141614.1	S-adenosylmethionine synthase 3-like (SAMS3L)	GACAAGACCATATTCCACCTCAAC	GCCTGCCTAACGATGTAAGC
XP_009107632.1	cytochrome P450 83B1 (CYP83b1)	TGAGATGAGGAAGATGTGTATGGT	ACAGTTGGTGAATGACAAGAGAAG
CDY12709.1	cytochrome P450 83A1 (CYP83A1)	GGCGATGCTTGAGGTTCC	GCTTGAGATGATCCGACTTGTG
XM_009138817.1	methylthioalkylmalate synthase 2 (MAM2)	CTATCGTTATGGCTTCGTCACTTC	CGTAGGGAGGGCAAGGAA
XM_009103756.1	S-alkyl-thiohydroximate lyase SUR1 (SUR1)	TCTCACGACCATCTCCACAA	TCCAGCCAATCTTCCATCCA
ACB59204.1	UDP-glycosyltransferase 74B1 (UGT74B1)	CGACGCATACTCCGAATCC	GGTGAGGTTGTTGGTGAAGAA
CDX75914.1	S-adenosylmethionine synthase 2 (SAMS2)	CTGAGCCGTTGTCTGTGTT	ACCATAAGCAGCAGTCTTCAAG
CDY37784.1	S-adenosylmethionine synthase 2-like (SAMS2L)	GTTCAGGTCTCTTACGCCATTG	TGTCCATACGCAGCAGTCT
DQ679980.1	homocysteine S-methyltransferase 1 (HMT1)	TTGGTGATGCTGAGTTTGAGAT	TTGATGGTGGAAGGTGTAGTC
CDX73052.1	cytosolic sulfotransferase 16-like (SULT16L)	GGACACTGGTGGCAAGAATG	AGCGAGAACGGTTGACGATA
XP_009106322.1	adenosylhomocysteinase 2-like (SAHH2L)	GCTCACCAAGGACCAATCTG	ACCCGAACCAACTCAAACAAT
AAP92453.1	adenosylhomocysteinase 1-like (LOC106311804), transcript variant X1 (SAHH1L-X1)	GCTGGTGCTAGAGTCATTGTG	GCGTTGTTCTTCATCTTCCTCAT
XP_009135865.1	adenosylhomocysteinase 2 (LOC106335699), transcript variant X4 (SAHH2L-X4)	CGTGATGTCGTGCTCGTT	CTTCTCGTCCAAGTGCTTAGG
XP_009114068.1	S-adenosylmethionine synthase 2-like (SAMS2L)	GAGGTGGTAATGGTAGGTTCTTG	CTTGTTAGACTTGAGTGGCTTCA

**Fig. 2:**
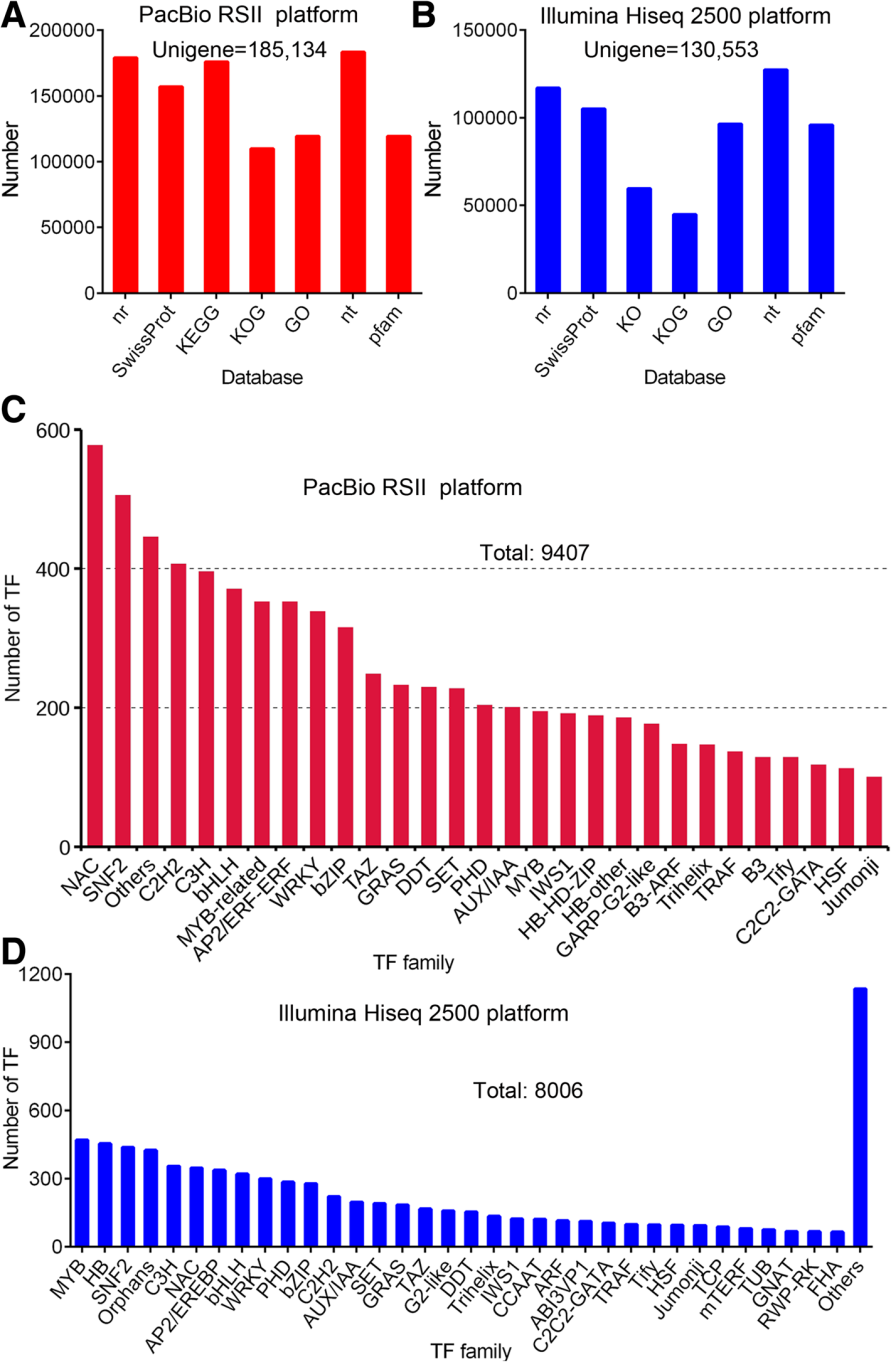
The unigene and transcription factor (TF) numbers produced by different sequencing platforms. **a** and **b**, the unigene numbers in transcriptome sequencing data generated from PacBio RSII platform and Illumina Hiseq sequencing platform, respectively. **c** and **d**, the TF numbers in PacBio RSII and Illumina transcriptome, respectively

In comparative analysis, we observed that the unigene number originated from PacBio RSII transcriptome (185,134 genes) was higher than that of Illumina transcriptome (130,553 genes; Table 1 and Fig. 2a and b), including 130,553 overlapping genes; the number of transfection factors (TFs) from PacBio RSII transcriptome was 9407, which was higher than 8006 from Illumina (Table 1; Fig. 2c and d).

### Functional annotation of unigenes

The GO, KEGG, and KOG functional annotations of unigenes in PacBio RSII transcriptome were identical to unigenes in Illumina transcriptome (Additional file 3 Figure S3). GO classification showed most unigenes were associated with cellular and metabolic processes with the molecular functions of binding and catalytic activity (Additional file 3 Figure S3A). KEGG and KOG classification showed that the top clusters involved unigenes were energy and carbohydrate metabolism, transport, catabolism and signal transduction (Additional file 3 Figure S3B and C). Annotation against NR database showed that unigenes in PacBio transcriptome were identical to *Brassica oleracea* (48.4%), followed with *Brassica napus* (38.0%) and *Brassica rapa* (3.0%), while the unigenes in Illumina transcriptome were identical to *Brassica napus* (63.0%) followed with *Brassica rapa* (19.3%; Additional file 3: Figure S3D).

### Identification of differentially expressed genes (DEGs) in response to different stimuli

**Fig. 3:**
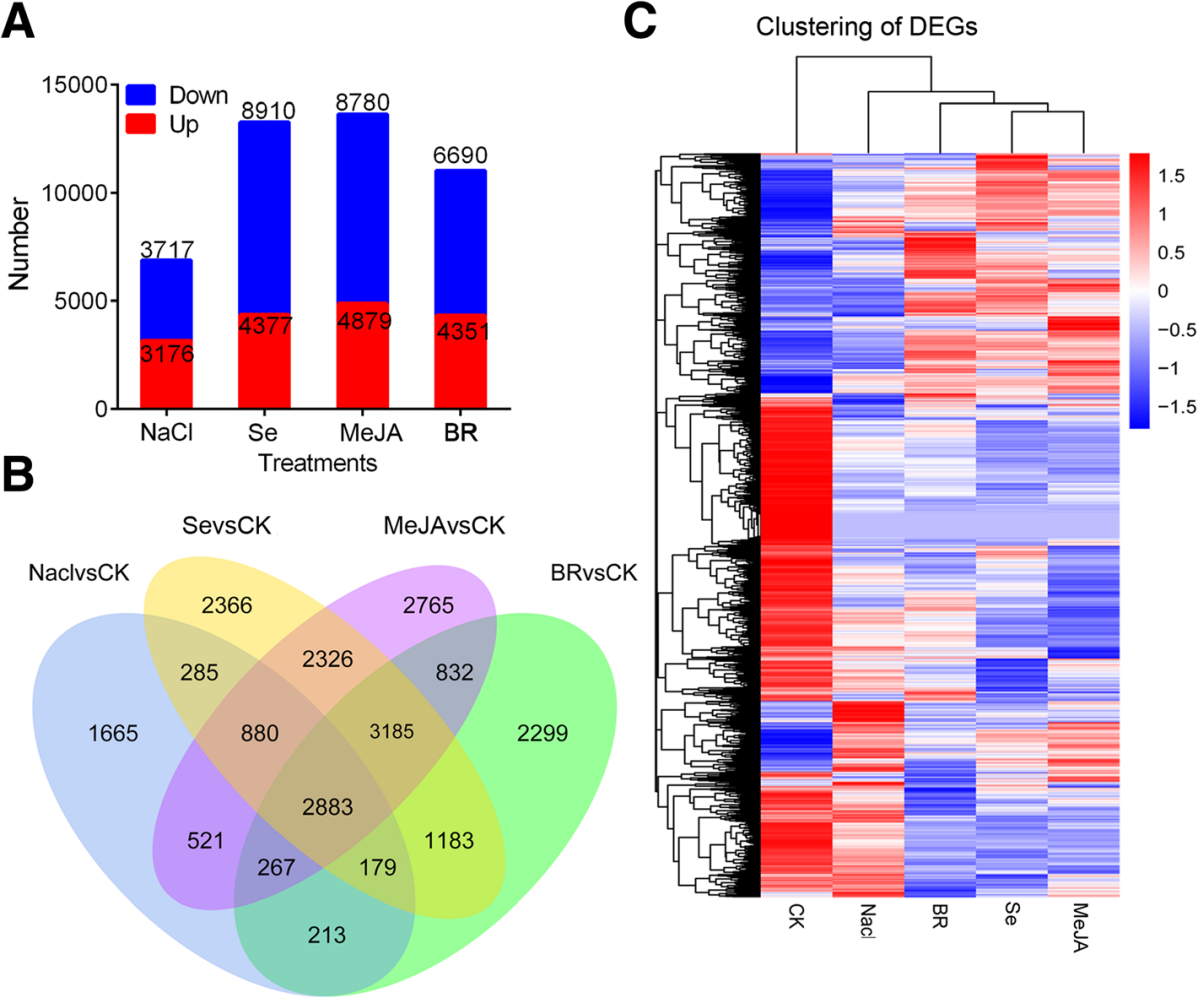
The distribution of differentially expressed genes (DEGs) induced by different treatments. **a** and **b**, the distribution of DEGs induced by different treatments and the venn figure, respectively. **c** clustering analysis of the DEGs. Blue, downregulated. Red, upregulated. MeJA, methyl jasmonate; Se, sodium selenite, BR, brassinolide

DEGs induced by different stimuli were identified from combined data of Illumina transcriptome with PacBio RSII transcriptome. A total of 6893 (including 3176 up- and 3717 down-regulated DEGs), 13,287 (including 4377 up- and 8910 down-regulated DEGs), 13,659 (including 4879 up- and 8780 down-regulated DEGs), and 11,041 DEGs (including 4351 up- and 6690 down-regulated DEGs) were identified from *Chinese kale* seedlings under NaCl, Se, MeJA, and BR treatment, respectively (*p*-value ≤0.05, log2FC ≥ 1 or ≤ − 1; Fig. 3a). A total of 2883 genes, including 685 up- and 2187 down-regulated DEGs, were simultaneously dysregulated by all NaCl, Se (sodium selenite 100 μM), MeJA (40 μM), and brassinolide (BR, 1.5 μM) treatments (Fig. 3b). The heatmap clustering of these DEGs is shown in Fig. 3c.

### GO and KEGG pathway enrichment analysis

Functional enrichment analysis showed NaCl, BR, MeJA, and Se-induced DEGs were associated with 26 ~ 135 GO terms including biological process terms of indole-containing compound metabolic process, cellular glucan metabolic process, carbohydrate metabolic process, and polyol biosynthetic process; cellular component terms of Holliday junction resolvase complex and external encapsulating structure; and molecular function terms of hydrolase activity of hydrolyzing O-glycosyl compounds, calcium ion binding, xyloglucan-xyloglucosyltransferase activity, and polysaccharide metabolic process (Additional file 4: Table S1). These DEGs were significantly involved in KEGG pathways including starch and sucrose metabolism, limonene and pinene degradation, stilbenoid, diarylheptanoid and gingerol biosynthesis, phenylalanine, tyrosine and tryptophan biosynthesis, valine, leucine and isoleucine biosynthesis, cysteine and methionine metabolism, and glucosinolate biosynthesis (Additional file 5: Table S2). The DEGs induced by MeJA, BR, and Se treatments were associated with plant-pathogen interaction pathway, and Se-induced DEGs were associated with the biosynthesis of glucosinolates (Additional file 5: Table S2 and Additional file 6: Figure S4). The KEGG pathways associated with DEGs induced by four stimuli are shown in Additional file 5: Table S2.

### Analysis of DEGs in pathways related to glucosinolate biosynthesis

**Fig. 4:**
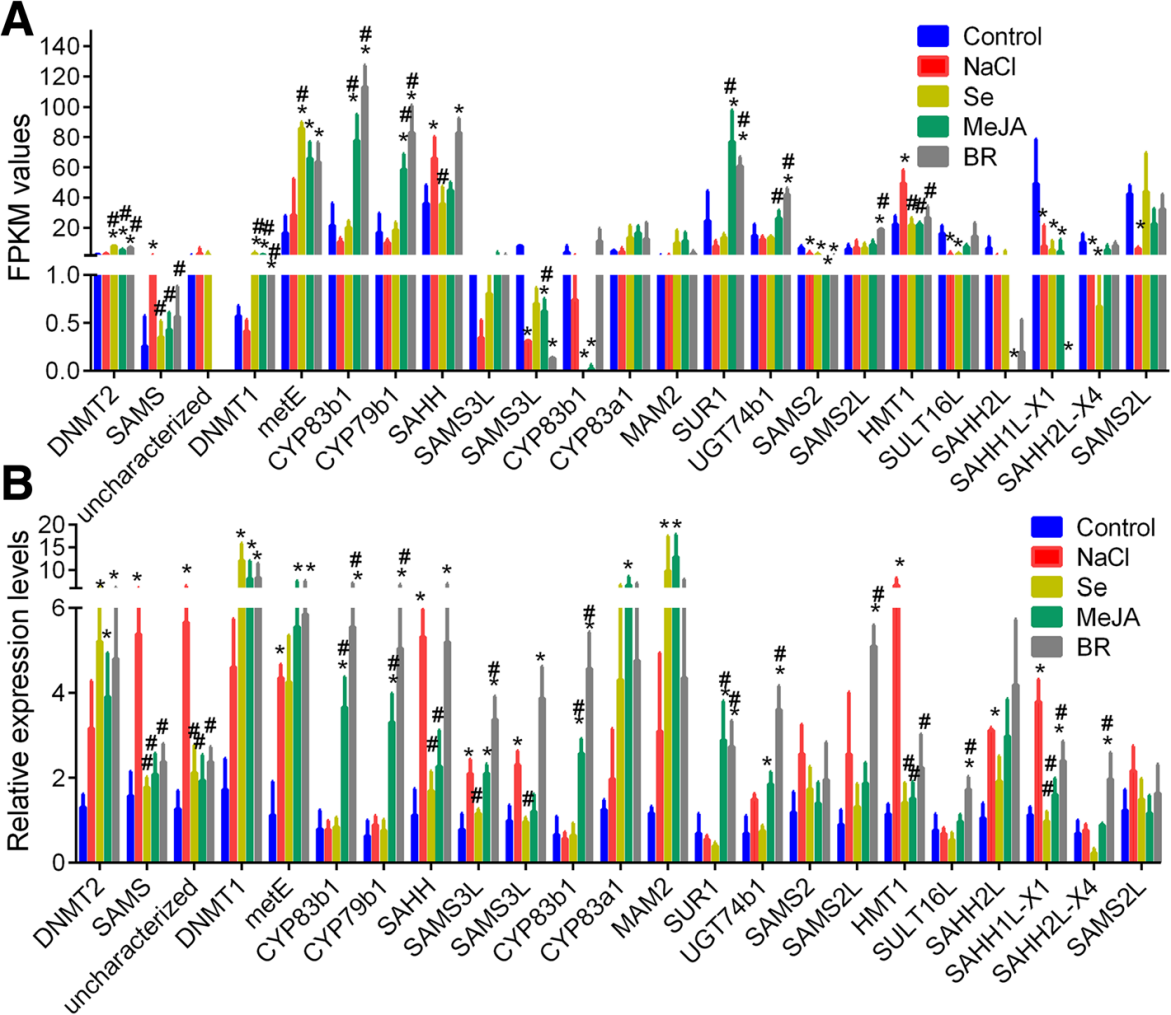
The FKPM values and relative levels of 23 genes related to glucosinolate biosynthesis. **a** the FKPM values of 23 genes in samples treated with different treatments by sequencing. **b** the relative expression levels of 23 genes determined by qRT-PCR. * notes *p* < 0.05 vs. control, and # notes *p* < 0.05 vs. NaCl, respectively. MeJA, methyl jasmonate; Se, sodium selenite, BR, brassinolide

According to aforementioned results, we confirmed that the influence of NaCl, BR, MeJA, and Se treatments on glucosinolates accumulation might be mediated by altering the expression profiles of sulforaphane and/or glucosinolate biosynthesis related genes. Accordingly, we found the FPKM (fragments per kilobase of transcript per million mapped reads) values of 23 unigenes related to glucosinolate biosynthesis were dysregulated by NaCl, BR, MeJA, and Se treatments (Fig. 4a). The mRNA relative expression levels profiles of most genes by qRT-PCR were similar to that of FPKM values by sequencing (Fig. 4a and b).

Sequencing and qRT-PCR analysis confirmed the expressions of SAMS (S-adenosylmethionine synthase; CDY40532.1), SAHH (adenosylhomocysteinase 1; CDY01177.1) and HMT1 (homocysteine S-methyltransferase 1; DQ679980.1) were upregulated by NaCl treatment; the expression of DNMT1 [(cytosine-5)-methyltransferase CMT1; CDY19364.1], DNMT2 (CDX99400.1) and metE (5-methyltetrahydropteroyltriglutamate:homocysteinemethyltransferase 1; CDX85436.1) were increased by Se, MeJA and BR treatments; the expression of CYP83b1 (CDX80735.1 and XP_009107632.1), CYP79b1 (CDX69373.1), SUR1 (XM_009103756.1) and UDP-glycosyltransferase 74B1 (UGT74b1; ACB59204.1) were upregulated by MeJA and BR (*p* < 0.05, Fig. 4a and b). Different expression pattern of SAMS3L (XP_009141614.1), CYP83b1 (XP_009107632.1), CYP83a1 (CDY12709.1), MAM2 (methylthioalkylmalate synthase 2; XM_009138817.1), SAHH1L-X1 (adenosylhomocysteinase 1-like (LOC106311804), transcript variant X1; AAP92453.1) and SAHH2L X4 (adenosylhomocysteinase 2 (LOC106335699), transcript variant X4; XP_009135865.1) might due to the loss assembled reads by sequencing. The consistency between sequencing and PCR analyses of 17 genes (73.91%, 17/23) showed the high confidence of sequencing data. The glucosinolate biosynthesis pathway and DEGs induced by Se, NaCl, MeJA and BR are shown in Additional file 6: Figure S4. The key genes involved in glucosinolate biosynthesis was the desulfoglucosinolate sulfotransferase (SOT) A/B/C (ST5a_b_c), which were downregulated by all treatments (Additional file 7: Figure S5), suggesting the impact of different treatment on glucosinolate biosynthesis.

### Dysregulated TFs

**Fig. 5:**
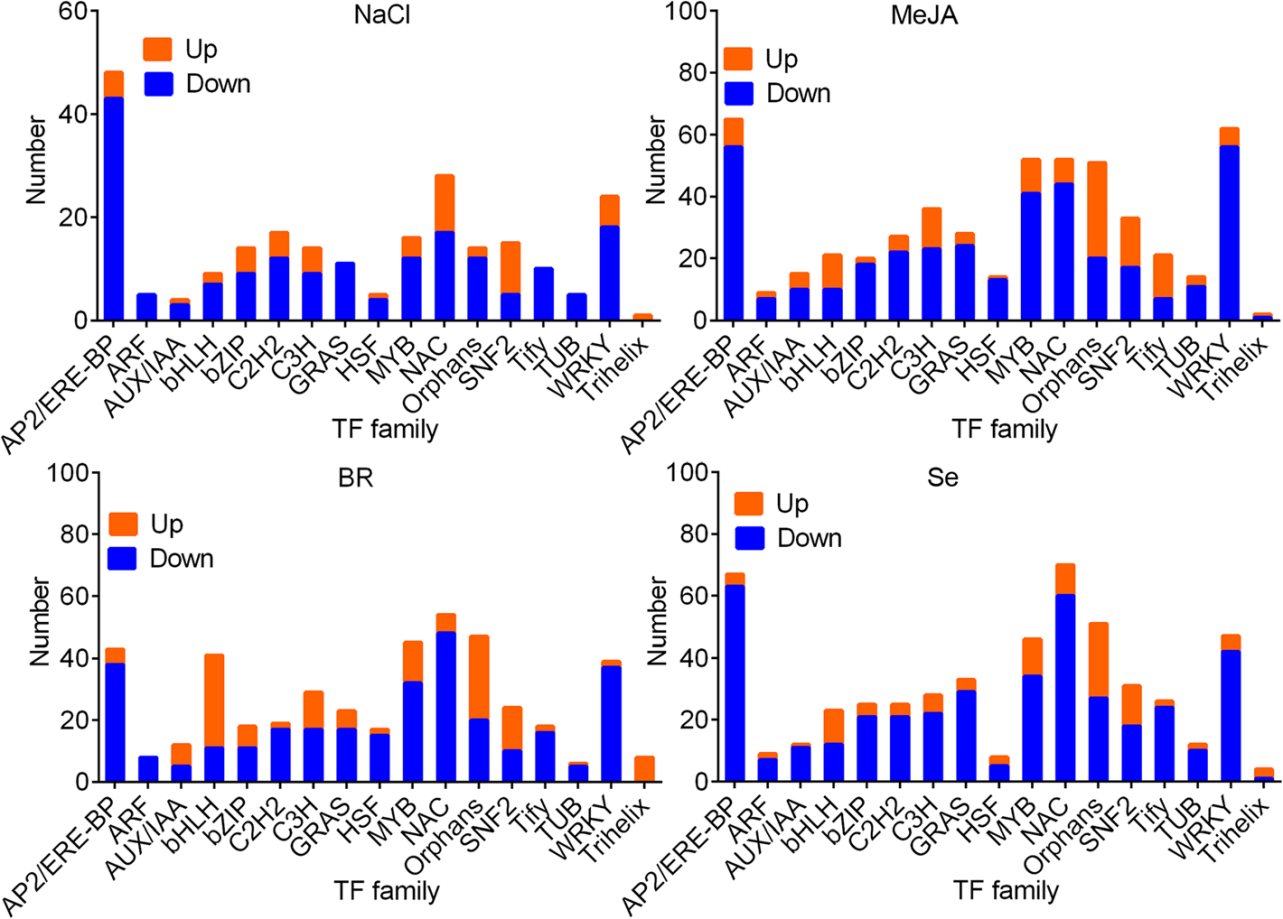
Dysregualted transcription factor (TF) numbers by four different treatments. Blue, downregulated TFs. Orange, upregulated TFs

We further confirmed that NaCl, MeJA, BR and Se treatment downregulated the members in MYB family (including MYB1R1, MYB44, WRKY45 and MYB24), AUX/IAA family (IAA9, IAA11 and auxin response factor 2, ARF2), NAC family (NAC-domain protein 5–11, NAC5–11; NAC55, ATAF2, and NAC29), AP2-EREBP family members (ERF19, ERF113, RAP2–13, and ERF12), SNF2 family, and WRKY family (including WRKY33, WRKY18, WRKY48, WRKY51, WRKY22–1), and upregulated MYB28 and WRKY47. Most TFs were downregulated by treatments (Fig. 5).

## Discussion

Glucosinolates are a group of sulfur-rich anionic products naturally generated in widely consumed cruciferous vegetables. We performed the combined transcriptome analysis of NGS and SMRT sequencing and investigated the molecular mechanism of sulforaphane and glucosinolate biosynthesis in Chinese kale seedlings in response to abiotic stresses. The high error rate in long reads generated from PacBio platform was corrected by short reads produced by Illumina sequencing. We found SMRT transcriptome contained 185,134 unigenes, that was higher than 130,553 unigenes from Illumina transcriptome, suggesting higher performance of SMRT sequencing compared with NGS.

The combination of NGS short reads and SMRT long reads identified 6893, 13,287, 13,659 and 11,041 DEGs in kale seedlings in response to NaCl (75 mM), MeJA (40 μM), Se (100 μM) and BR (1.5 μM) treatment, respectively. KEGG pathway enrichment analysis showed these genes were associated with energy metabolism, biosynthesis of amino acids including phenylalanine, tyrosine, tryptophan, phenylalanine, cysteine, methionine, valine, leucine and isoleucine, and glucosinolate (Additional file 5: Table S2). These pathways were core pathways for the synthesis of glucosinolates (Additional file 6: Figure S4). These facts showed that these biosynthetic pathways were influenced by NaCl, MeJA, Se and BR treatments in Chinese kale seedlings, and the glucosinolates might take important roles in the plant defense or resistance to these abiotic stresses.

In stressed or specific conditions, glucosinolates are released from vacuoles and are hydrolyzed by MYO to unstable thiohydroximate-O-sulfonate, which spontaneously converts to isothiocyanates including sulforaphane [17, 29]. Glucosinolates are grouped into three groups, including aliphatic, aromatic and indolic glucosinolates. The tryptophan amino acid is the substrate of indolic glucosinolates, and methionine is the substrate of aliphatic glucosinolates [13, 17]. The subsequently conversion of tryptophan-derived indolic glucosinolates and methionine-derived aliphatic glucosinolates to S-alkylthiohydroximates (SAM) is catalyzed by CYP83b1 and CYP83a1 [30]. Next, SAMs are conversed into thiohydroximates, desulfoglucosinolates and sulfated products by SUR1, UGT74B1 and ST5b_c, respectively, in step-by-step ways [30–32]. We confirmed that the content of aliphatic glucosinolates was highest in Chinese kale seedlings, followed with indolic glucosinolates and then aromatic glucosinolates. In addition, we confirmed that the content of aliphatic glucosinolates was increased by MeJA and BR treatments, while aromatic glucosinolates was increased by Se and BR treatments (Fig. 3). Transcriptome analysis showed that the expression of genes in core pathways of aliphatic (including CYP83a1, SUR1, and UGT74b1) and aromatic glucosinolates (including CYP83b1, SUR1 and UGT74b1), were increased by MeJA and BR treatments (Fig. 4 and Additional file 6: Figure S4). In addition, we found the contents of sulforaphane and total glucosinolates were increased by MeJA and BR treatments compared with control. The expressions of CYP83b1, SUR1 and UGT74b1 were upregulated followed MeJA treatment (Fig. 4). Although NaCl treated decreased the content of aliphatic and indolic glucosinolates and sulforaphane, the expression of the aforementioned core genes did not decrease by NaCl treatment. This might due to the decreased expression of ST5a_b_c by NaCl treatment (Additional file 7: Figure S5). We also observed the decrement in ST5a_b_c expression by Se, MeJA and BR. It has been reported that the synthesis of aliphatic glucosinolates could be controlled by ST5b_c, and the aromatic glucosinolates is controlled by ST5a (Additional file 6: Figure S4). These demonstrated the complex and ordinal mechanism of glucosinolates biosynthesis in plant [33].

ST5a_b_c, are three desulfoglucosinolate SOTs that catalyze the final step in glucosinolate biosynthesis by catalyzing different substrate specificities and producing S-containing products [34, 35]. The most important glucosinolate, sulforaphane, is a sulfur-rich hydrolysis product of aliphatic (methylseleno)glucosinolate, the methionine-derived glucosinolates [36–38]. Our current study confirmed that the contents of all glucosinolates and sulforaphane in Chinese kale were decreased by NaCl stress (75 mM). This was consistent with the results from the study by Esfandiari et al. [17]. Esfandiari et al. showed that NaCl stress (80 and 160 mM) decreased the content of glucosinolates in broccoli via suppressing the expression of CYP83a1 and CYP83b1 [17]. They also suggested that the increased contents of sulforaphane by NaCl stress (80 and 160 mM) were associated with upregulation of MYO and ESM1. Guo et al. showed the higher expression of MYO was found in Chinese kale roots with low-glucosinolates [25], which might responsible for the increased sulforaphane [17]. Our study demonstrated that the expression of CYP83a1 and CYP83b1 were not significantly influenced by 75 mM NaCl stress. In addition, the treatment with 75 mM NaCl decreased the contents of both sulforaphane and glucosinolates. Although total and the major aliphatic glucosinolates were increased by MeJA and BR treatments, the FPKM of ST5a (crucial for aromactic glucosinolates) and ST5b_c (crucial for aliphatic glucosinolates; Additional file 6: Figure S4) coding sequences were decreased by MeJA and BR compared with control (Additional file 7: Figure S5). These differences might due to the plant variety, tissues or growth conditions [17, 27, 39].

Other studies of Chinese kale transcriptome showed that the expression of CYP83a1 and CYP83b1 showed high expression level in root [24], and in plants with low contents of glucosinolates [25]. It has been reported that the biosynthesis of glucosinolates in susceptible varieties of Chinese cabbage were active than that in resistant varieties, and glucosinolates were increased by infection of *Plasmodiophora* [39], while that in resistant varieties was not influenced by treatments. They also showed that the two susceptible varieties had different contents of indolic and aliphatic glucosinolates in leaves or roots, while aromatic glucosinolates were the major glucosinolates in resistant varieties in response to *Plasmodiophora*, which were differentially influenced by the addition of SA and JA [39]. Our study was performed with 7-day old (post germination) Chinese kale seedlings, and the whole plant contents of glucosinolates were detected. The distribution of glucosinolates was averaged then, and these might also be the reasons for the differences between our results with others from Ludwig-Müller et al. [40], Esfandiari et al. [17] and others.

## Conclusion

In summary, we performed the de novo assembly of Chinese kale genomes using SMRT sequencing combined with NGS sequencing. DEGs induced by NaCl, MeJA, BR and Se treatment were identified using combination of NGS and SMRT transcriptome. MeJA and BR treatments increased the content of total glucosinolates and/or sulforaphane by upregulated the expression of genes related to glucosinolate core pathways, including CYP83b1, CYP83b1, SUR1 and/or UGT74b1. We also confirmed that NaCl (75 mM) inhibited the biosynthesis of sulforaphane, tryptophan-derived indolic glucosinolates and methionine-derived aliphatic glucosinolates, and MeJA treatment enhanced them. We concluded that the treatment of MeJA, Se and BR, especially MeJA, confer the formation and biosynthesis of glucosinolates and sulforaphane.

## Methods

### Plant materials and preparation

High sulforaphane Chinese kale germplasm resource BOK92 (inbred line) was obtained from the seed bank of College of Horticulture and Landscape, Hunan Agricultural University, Changsha, China. Seeds were sawed in nutrient soils (silica sands, sermiculite). After germination, seedlings were managed with standard nutrient solutions and treated with NaCl (0, 25, 50, 75, 100, 125 and 150 mM), MeJA (0, 20, 40, 60, 80, 100 and 120 μM), Se (0, 20, 40, 60, 80, 100 and 120 μM) and BR (0.0, 0.5, 1.0, 1.5, 2.0, 2.5 and 3.0 μM) with four triplicates of 30 seeds each. Control group was treated with standard nutrient solutions only. Experiments were performed in tissue culture room of Hunan Agricultural University at 22 °C ~ 25 °C, 75% relative humidity, 35,000 lx illumination intensity, and a 16 L: 8 D light cycle, with two handle irradiation per day. At 7-day post germination, seedlings were collected, snapped in liquid nitrogen, and stored at − 80 °C before RNA isolation.

### Extraction and measurements of sulforaphane and glucosinolate

Sulforaphane was extracted from frozen samples of kale seedlings. Samples were grinded into powder and then immersed into purified water for 2 h at room temperature, followed with dichloromethane extraction for 10 min. Sodiumfulfate was used for drying. Extractions were then firstly filtered, vacuum dried, eluted using acetonitrile, and finally filtered (0.22 μm). Sulforaphane standard was purchased from Shanghai yuanye Bio-Technology, China, (CAS No. 4478-93-7, Art. No.G18O8C45982). The content of sulforaphane was determined using high performance liquid chromatography (HPLC: Shimadzu, Kyoto, Japan) methods with conditions as follows: C18 column (250 mm × 4.6 mm, 5 μm, at 30 °C), mobile phase (20% acetonitrile-80% water) at speed of 1 mL/min, retention time 20 min, and wavelength 202 nm.

Glucosinolate were methanol-extracted from kale seedlings. Grinded powder was extracted with 70% methanol at 75 °C for 20 min, with addition of sinigrin (in water, 0.5 mg, internal standard). After the glucosinolate extraction became cold, barium acetate was added, followed with centrifugation. The supernatants were then extracted with 70% methanol and centrifuged for supernatants. Desulfoglucosinolates were isolated using anion-exchange chromatography. DEAESephadex A-25 was pretreated with acetic acid and then filled with resin. Crude extract was eluted with water and desulfoglucosinolates were eluted using sulfatase solution at room temperature overnight. Desulfitation glucosinolates were collected using water-elution. Desulfoglucosinolates were separated using chromatography with gradually increased methanol concentrations [5% methanol for 1 min, linear gradient methanol (5%~ 100%) for 8 min, 100% methanol for 2 min, and 5% methanol for 2 min]. The contents of desulfoglucosinolates were determined using ULPC-MS technology [diode array detector (229 nm), C18 column (100 × 2.1 mm), mobile phase of 75% methanol-25% water] and mass spectrometer. Glucosinolates standards were purchased from (Sigma-Aldrich, GER, CAS No. 3952-98-5, Art. No. S1647-500MG).

### RNA extraction and transcriptome sequencing

Altered genomic expression profiles in response to different stimulation (NaCl, MeJA, SE and BR) were identified using the NGS. Total RNA was isolated from kale seedlings using TRIzol (Invitrogen, USA) and treated with RNase-free DNase I (Takara, Japan). After quality assessment using Agilent 2100 Bioanalyzer (Agilent Technologies, USA) and NanoDrop2000c spectrophotometer (NanoDrop products, USA), RNA samples were used for the synthesis of the first strand cDNA using a SMARTer PCR cDNA Synthesis Kit (Clontech Laboratories, USA). PCR optimization followed with large scale PCR were conducted for Illumina sequencing (the NGS, Hiseq PE 150) and the PacBio RSII sequencing (the third generation sequencing). Fifteen libraries were constructed and subjected to the NGS using Illumina sequencing platform and third-generation sequencing using the Pacific Biosciences RSII sequencing platform.

### Data processing

The NGS data were collected from Illumina sequencing platform and processed as usual. Data quality estimators, including base error rate (%), phred score (Q_30_), GC content (%), and clean data rate (%), was evaluated. Sequence data (CCS) containing full length and non-full length reads were generated from PacBio RSII platform and were processed using the SMRTlink 4.0 software (min Passes = 1, min Predicted Accuracy =0.8). Non-full length and full-length fasta files were then fed into the cluster step (isoform-level clustering, ICE). Hiseq reads in full-length transcripts were adjusted using proovread software [41] and used as the reference sequences in this study. Redundancy in the adjusted transcripts was removed using CD-HIT-EST program and unigenes were obtained subsequently.

### Gene functional annotation, CDS predication, analysis or SNP and SSR

Nr, Nt, Pfam, KEGG, KOG, GO, Swiss-Prot, and KO databases were visited for gene function of the unigenes. Open reading frame (ORF) corresponded to the unigenes were identified with basting against NR and Swissprot proteins databases. Estscan software (v3.0.3) [42] was used for remedial prediction of ORF in unigenes. Single nucleotide polymorphisms and insertion-deletions of the transcriptome were called using GATK3 (v3.2, QUAL< 30.0 and QD < 5.0) [43]. SSRs of the transcriptome were identified using MISA (v 1.0, http://pgrc.ipk-gatersleben.de/misa/misa.html).

### Identification of DEGs

Clean reads assembled using Trinity (version trinityrnaseq-2.0.2) [44], and assembled transcriptome was used for reads mapping by RSEM software [45]. Accordingly, FPKM levels of unigenes were obtained and used for calculation of relative expression level. DEGs were identified using the DESeq R package (v1.10.1) [46], with the criteria of adjusted *p*-value ≤0.05, and log2 (fold change) ≥ 1 (up) or ≤ − 1 (down). Cluster analysis of DEGs was performed using heatmap clustering.

### GO and KEGG pathway enrichment analysis

GOseq analysis (http://www.geneontology.org/) was conducted for GO functional classification analysis [47]. The KEGG pathways associated with DEGs were identified using KEGG-Based Annotation System (KOBAS) server (http://kobas.cbi.pku.edu.cn/home.do) [48]. Called GO terms and KEGG pathways with corrected *p* value (BH) < 0.05 were considered as significantly items.

### Identification of TFs and lncRNAs

TF among the DEGs were predicted using iTAK software (v1.2). LncRNAs of the transcriptome were identified using Coding-Non-Coding-Index (CNCI, default parameters), Coding Potential Calculator (CPC, e-value ≤1e-10), and Pfam database (default parameters).

### QRT-PCR analysis

Isolated total RNA from kale seedlings were reversely transcribed into the first-strand cDNA using Bestarq PCR RT Kit (DBI Bioscience, Germany). Primers were synthesized by Sangon (Shanghai, China) and were listed in Table 2. DNA template (1 μg) was used for PCR amplification using specific primers synthesized by Sangon (Shanghai, China, Table 1). Amplification (20 μL) was performed using Sybr Green qPCR master Mix (GENEray, GK8020) on an ABI 7500 fast real time PCR machine (ABI, USA). The reaction conditions were as follows: 94 °C for 3 min, 40 cycles of 94 °C 20 s, 58 °C 20 s, 72 °C 20 s. Relative expression levels of genes were analyzed using 2^- △△Ct^ methods. The 18 s gene was used as internal normal control gene in this study.

### Statistical analysis

All data of qRT-PCR analysis and contents of sulforaphane and glucosinolate were presented as mean ± standard deviation. All experiments were repeated at least three times. Statistics were performed using GraphPad Prism 6.0. One-way ANOVA was used to compare the data between groups with Holm-Sidak post hoc test. *P* < 0.05 was considered statistically significant.

## Additional files

Additional file 1: **Figure S1.** Comparison of PacBiosubreads quality and corrected reads. (TIF 1683 kb)

Additional file 2: **Figure S2.** The analysis of the length distribution of CDS, PacBio reads and lncRNA number. A, the length distribution of blast and Estscan-predicted CDS in unigenes in Illumina sequencing data, respectively. B and C, the length distribution of blast CDS in unigenes, Subreads, and consensus reads in PacBio sequencing data, respectively. D, the venn figure of lncRNA number predicted in different softwares. E and F, the distribution of SSR motifs in transcriptome generated from different platform. (TIF 1145 kb)

Additional file 3: **Figure S3.** Gene functional annotation of unigenes in transcriptome data from different platform. A-D, GO, KEGG, KOG, and NR classification of unigenes in transcriptome data from PacBio platform (Left) and Illumina (Right), respectively. (TIF 1729 kb)

Additional file 4: **Table S1.** Significant GO classifications associated with dfifferentially expressed genes (DEGs) induced by different treatments. (XLSX 754 kb)

Additional file 5: **Table S2.** Significant KEGG pathways associated with dfifferentially expressed genes (DEGs) induced by different treatments. (XLSX 72 kb)

Additional file 6: **Figure S4.** The glucosinolate biosynthesis pathway and related DEGs induced by sodium selenite treatment. Red and yellow boxes indicate upregulated DEGs, and green notes downregulated DEGs. (TIF 2356 kb)

Additional file 7: **Figure S5.** The FPKM level of ST5a_b_c under different treatments. * and ** notes *p* < 0.05 and 0.01 vs. control, respectively. # notes *p* < 0.05 vs. NaCl. (JPG 80 kb)

## Abbreviations

BR: Brassinolide
CCS: Circular consensus sequences
CNCI: Coding-Non-Coding-Index
CPC: Coding potential calculator
DEG: Differentially expressed genes
FLNC: Full-length non chimera
GO: Gene Ontology
GT: Glucuronyltransferase
IL-1β: Interleukin-1β
KEGG: Kyoto Encyclopedia of genes and genomes
KOG: Clusters of orthologous groups of proteins
LncRNAs: Long non-coding RNA
MAMs: Methylthioalkylmalate synthases
MeJA: Methyl jasmonate
NGS: Next-generation sequencing
NR: NCBI non-redundant protein sequences
Nrf2: Nuclear factor erythroid 2–related factor 2
NT: NCBI non-redundant nucleotide sequences
ORF: Open reading frame
SA: Salicylic acid
SAM: S-alkylthiohydroximates
Se: Sodium selenite
SMRT: Single-molecule real-time
SSR: Simple sequence repeat
SUR1: S-alkyl-thiohydroximatelyase or carbon–sulfur lyase
TF: Transcription factors
UGT74b1: UDP-glycosyltransferase 74B1
