# Supplementary figures and images for: Comparative transcriptome analyses of genes involved in sulforaphane metabolism at different treatment in Chinese kale using full-length transcriptome sequencing
Source: BMC Genomics. 2019 May 14;20:377. doi: 10.1186/s12864-019-5758-2 (PMC6518776; doi:10.1186/s12864-019-5758-2)

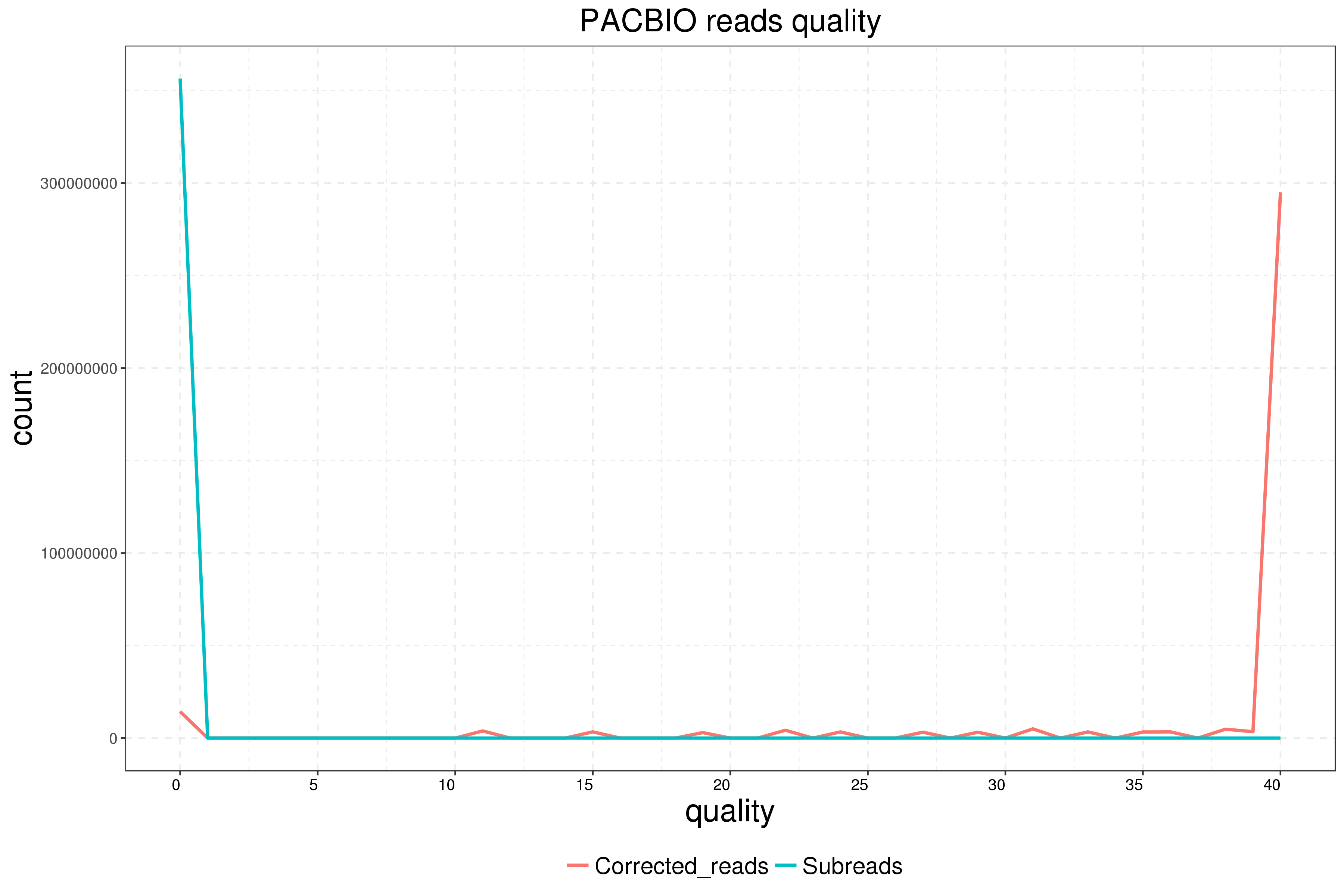

Supplement: Supplementary file 1 — Figure S1. Comparison of PacBiosubreads quality and corrected reads. (TIF 1683 kb) [file 12864_2019_5758_MOESM1_ESM.tif]

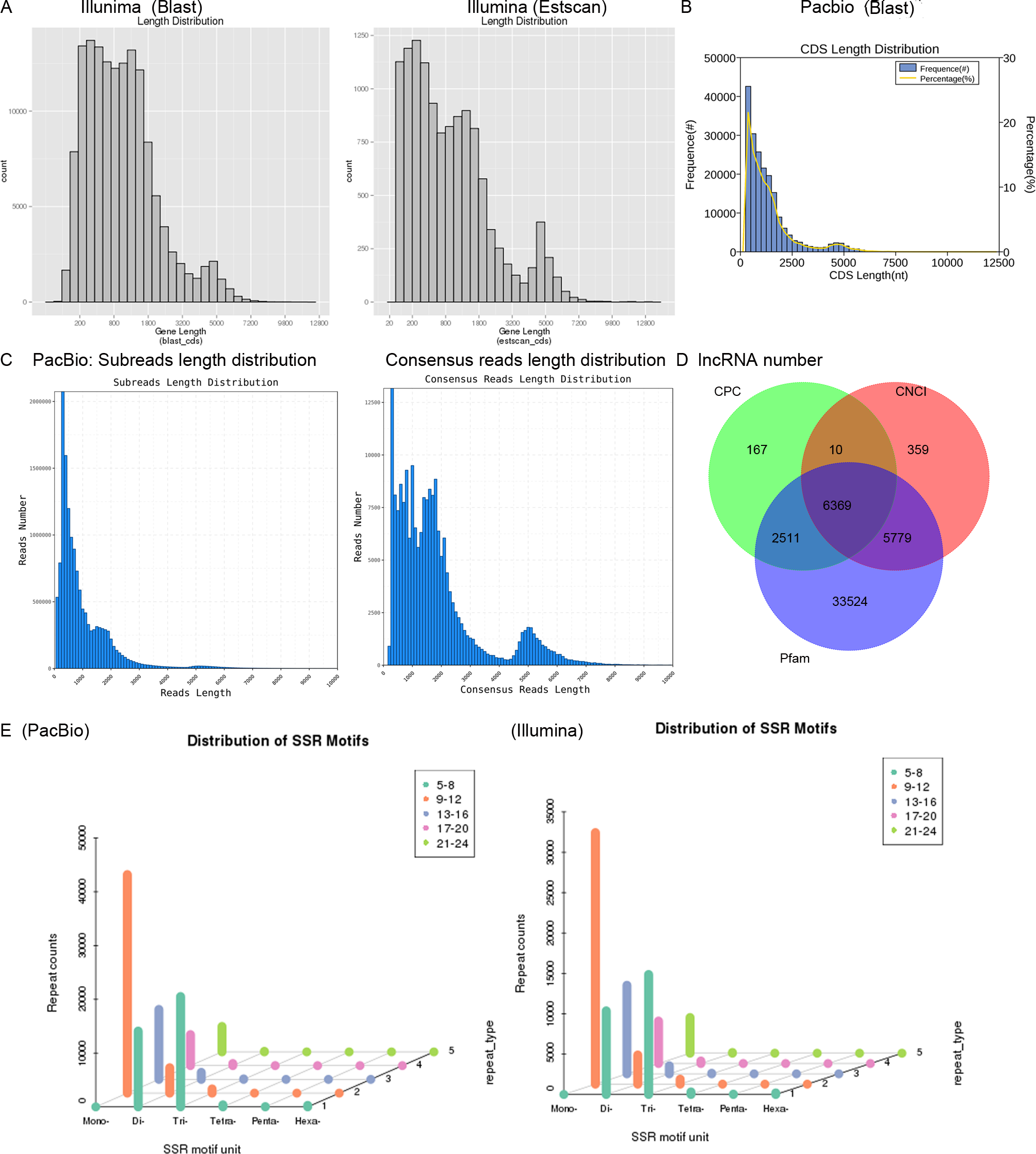

Supplement: Supplementary file 2 — Figure S2. The analysis of the length distribution of CDS, PacBio reads and lncRNA number. A, the length distribution of blast and Estscan-predicted CDS in unigenes in Illumina sequencing data, respectively. B and C, the length distribution of blast CDS in unigenes, Subreads, and consensus reads in PacBio sequencing data, respectively. D, the venn figure of lncRNA number predicted in different softwares. E and F, the distribution of SSR motifs in transcriptome generated from different platform. (TIF 1145 kb) [file 12864_2019_5758_MOESM2_ESM.tif]

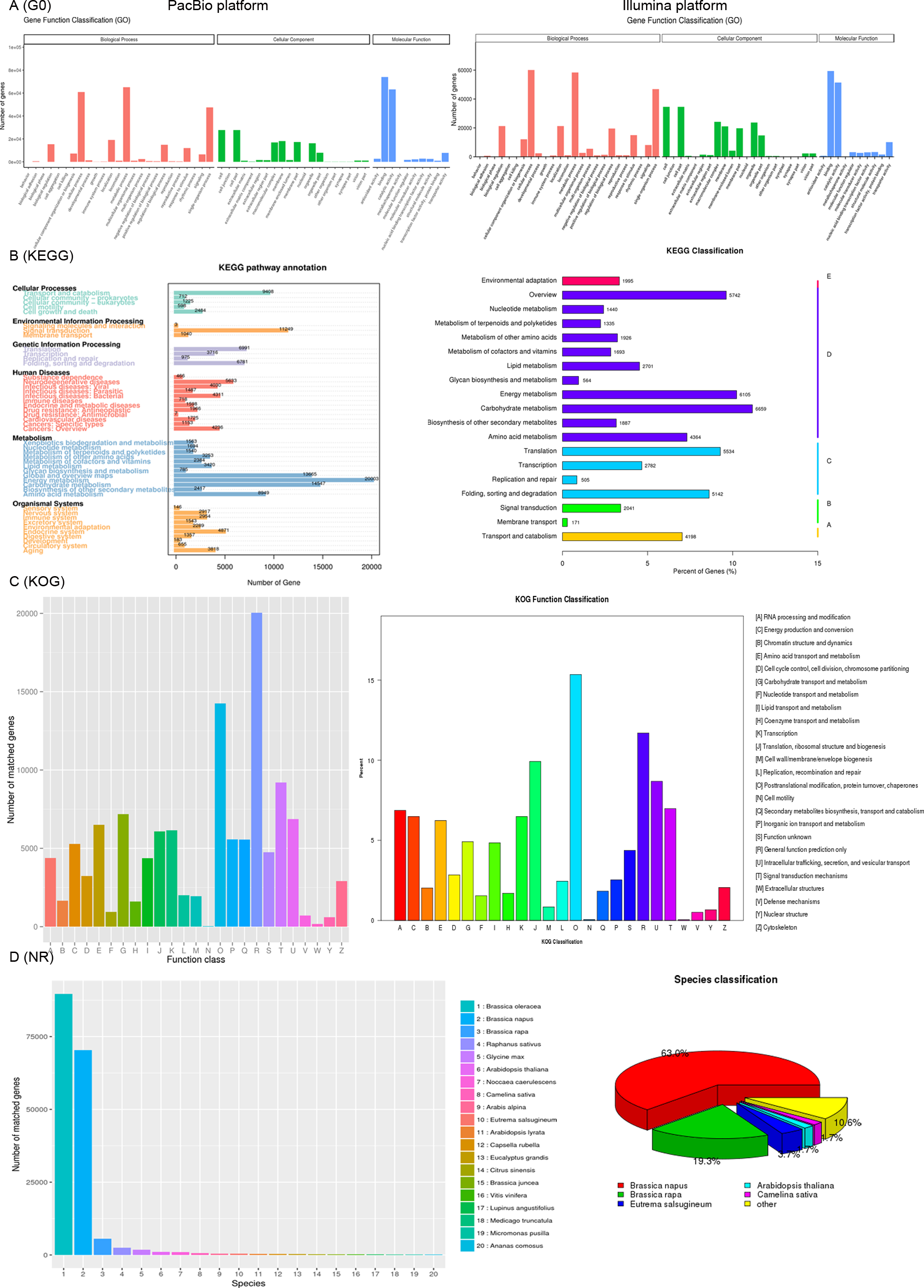

Supplement: Supplementary file 3 — Figure S3. Gene functional annotation of unigenes in transcriptome data from different platform. A-D, GO, KEGG, KOG, and NR classification of unigenes in transcriptome data from PacBio platform (Left) and Illumina (Right), respectively. (TIF 1729 kb) [file 12864_2019_5758_MOESM3_ESM.tif]

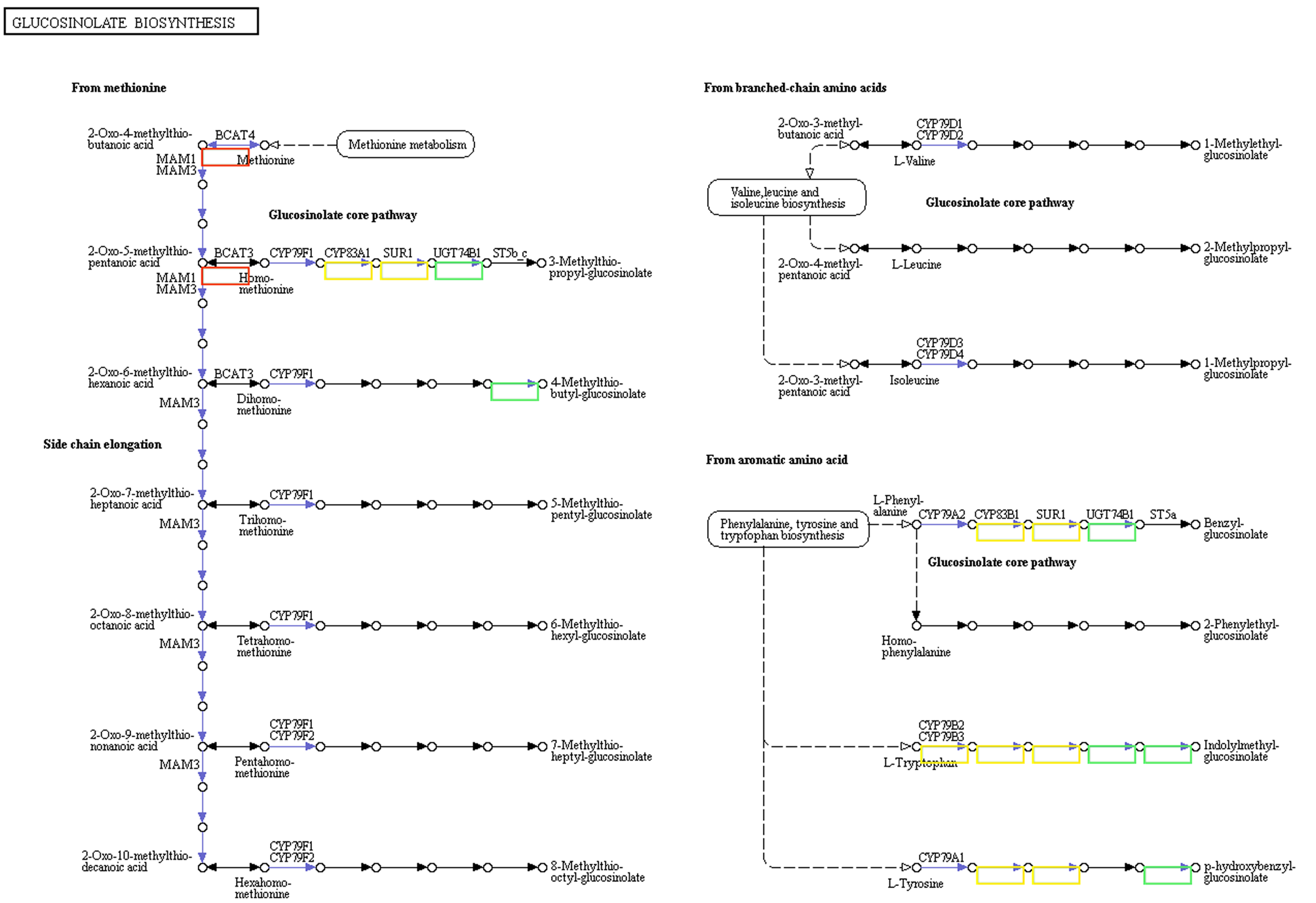

Supplement: Supplementary file 6 — Figure S4. The glucosinolate biosynthesis pathway and related DEGs induced by sodium selenite treatment. Red and yellow boxes indicate upregulated DEGs, and green notes downregulated DEGs. (TIF 2356 kb) [file 12864_2019_5758_MOESM6_ESM.tif]

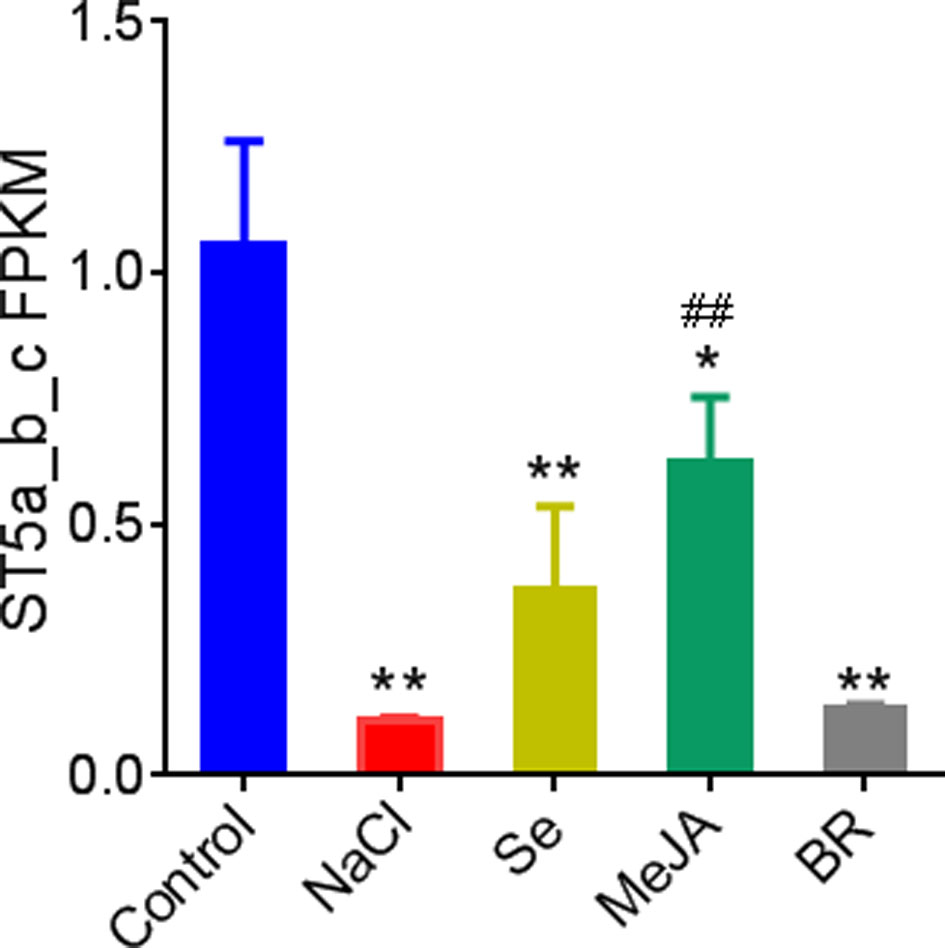

Supplement: Supplementary file 7 — Figure S5. The FPKM level of ST5a_b_c under different treatments. * and ** notes p < 0.05 and 0.01 vs. control, respectively. # notes p < 0.05 vs. NaCl. (JPG 80 kb) [file 12864_2019_5758_MOESM7_ESM.jpg]
